# Physician practices for withdrawal of medications in inactive systemic juvenile arthritis, Childhood Arthritis and Rheumatology Research Alliance (CARRA) survey

**DOI:** 10.1186/s12969-019-0342-5

**Published:** 2019-07-22

**Authors:** Susan Shenoi, Kabita Nanda, Grant S. Schulert, John F. Bohnsack, Ashley M. Cooper, Bridget Edghill, Miriah C. Gillispie-Taylor, Baruch Goldberg, Olha Halyabar, Thomas G. Mason, Tova Ronis, Rayfel Schneider, Richard K. Vehe, Karen Onel, for the Childhood Arthritis and Rheumatology Research Alliance Systemic Juvenile Idiopathic Arthritis Workgroup

**Affiliations:** 1 Department of Pediatrics, Division of Rheumatology, University of Washington School of Medicine & Seattle Children’s Hospital and Research Center, MA.7.110, 4800 Sand Point Way NE, Seattle, WA 98105 USA; 2 Division of Rheumatology, Cincinnati Children’s Hospital Medical Center and Department of Pediatrics, University of Cincinnati College of Medicine, Cincinnati, OH USA; 3 Division of Pediatric Rheumatology, University of Utah Hospital, Salt Lake City, UT USA; 4 Division of Pediatric Rheumatology, Children’s Mercy Kansas City, Department of Pediatrics, University of Missouri-Kansas City, Kansas City, MO USA; 5 Parent of systemic juvenile arthritis patient representative, Kansas City, USA; 6 Department of Pediatrics, Rheumatology, Levine Children’s Hospital/Carolinas Healthcare System, University, North Carolina, Chapel Hill, NC USA; 7 Department of Pediatrics, Division of Pulmonary Allergy Immunology and Rheumatology, University of Texas Health Science Center at Houston, Houston, USA; 8 Department of Pediatrics Boston Children’s Hospital, Division of Immunology, Boston, MA USA; 9 Departments of Medicine and Pediatrics, Mayo Clinic College of Medicine, Rochester, MN USA; 10 Division of Pediatric Rheumatology, Children’s National Health System, Department of Pediatrics, George Washington University School of Medicine and Health Sciences, Washington, DC USA; 11 The Department of Paediatrics, University of Toronto, Hospital for Sick Children, Toronto, Ontario Canada; 12 Division of Pediatric Rheumatology, Department of Pediatrics, University of Minnesota Medical School & University of Minnesota Masonic Children’s Hospital, Minneapolis, MN USA; 13 Division of Pediatric Rheumatology, Hospital for Special Surgery, Department of Pediatrics, Weill Cornell Medical College, New York, NY USA

**Keywords:** Systemic Juvenile Idiopathic Arthritis, Inactive disease, Withdrawal of medications, CARRA

## Abstract

**Background:**

We describe a Childhood Arthritis and Rheumatology Research Alliance (CARRA) survey of North American pediatric rheumatologists that assesses physician attitudes on withdrawal of medications in systemic juvenile idiopathic arthritis (SJIA).

**Methods:**

A REDCap anonymous electronic survey was distributed to 100 random CARRA JIA workgroup physician-voting members. The survey had three broad sections including: A) demographic information; B) physicians’ opinions on clinical inactive disease (CID) in SJIA and C) existing practices for withdrawing medications in SJIA.

**Results:**

The survey had an 86% response rate. 88 and 93% of participants agreed with the current criteria for CID and clinical remission on medications (CRM) respectively. 78% thought it necessary to meet CRM before tapering medications except steroids. 76% use CARRA SJIA consensus treatment plans always or the majority of the time. All participants weaned steroids first in SJIA patients on combination therapy, 47% waited > 6 months before tapering additional medications. 35% each tapered methotrexate over > 6 months and 2–6 months; however, 39% preferred tapering anakinra, canakinumab and tocilizumab more quickly over 2–6 months and favored spacing the dosing interval for canakinumab and tocilizumab. When patients are on combination therapy with methotrexate and biologics, 58% preferred tapering methotrexate first while others considered patient/family preference and adverse effects to guide their choice.

**Conclusion:**

Most CARRA members surveyed use published consensus treatment plans for SJIA and agree with validated definitions of CID and CRM. There was agreement with tapering steroids first in SJIA. There was considerable variability with tapering decisions of all other medications.

**Electronic supplementary material:**

The online version of this article (10.1186/s12969-019-0342-5) contains supplementary material, which is available to authorized users.

## Background

Systemic juvenile idiopathic arthritis (SJIA) comprises 10–15% of juvenile arthritis. SJIA is unique from other categories of JIA, due to its similarity to monogenic autoinflammatory diseases, systemic features and response to IL1 inhibitors. The introduction of anti-IL1 and anti-IL6 agents revolutionized the treatment of SJIA allowing the majority of patients to achieve inactive disease states [1].

SJIA can follow a monophasic (one episode of disease followed by remission), polyphasic (multiple flares of either systemic or arthritic features), or persistent (unremitting) course. Currently there are no genetic or immunologic clues that allow treating physicians to determine which of these three courses patients will follow over time. Hence, clinical decisions on when to continue, taper, or withdraw medications are often subjective and vary from physician to physician. Clinicians need to balance the risk of medication withdrawal causing disease flare versus the risk of continued medication exposure and related side effects.

Childhood Arthritis and Rheumatology Research Alliance (CARRA) is the largest pediatric rheumatology network across North America, committed to fostering research and innovation in pediatric rheumatologic diseases. This paper describes a CARRA survey conducted by the SJIA workgroup, to study current physician practices across North America, regarding the definition of inactive disease in SJIA, and withdrawal patterns of medications in SJIA once inactive disease is reached.

## Materials and methods

CARRA membership has grown exponentially since inception and as of June 2017, it has 555 members across 119 sites in United States and Canada. The JIA workgroup consists of 117 members. The SJIA workgroup consists of 62 pediatric rheumatologists.

At the 2015 CARRA SJIA workgroup meeting (Austin, Texas) there was a unanimous decision to conduct a survey examining current attitudes and trends amongst pediatric rheumatology physicians for withdrawal of medications in inactive disease in SJIA. REDCap is a secure web-based application that facilitates building and housing online surveys [2]. A cross-sectional anonymous electronic survey using REDCap was distributed to 100 randomly selected physician-voting members of the CARRA JIA workgroup. Seattle Children’s Hospital (STUDY00000532) institutional review board (IRB) and Hospital for Special Surgery (2017–0276) approved the study. The study was deemed exempt by the IRB and consent was consent was not required.

Additional file 1: At the 2016 CARRA SJIA workgroup meeting (Toronto, Ontario), face-to-face input from group members (*n* = 63, 2 were parent representatives including BE, coauthor) was obtained to develop several fields, questions and case scenarios for the survey, following which a smaller core group refined and finalized the survey. CARRA leadership and Protocol Evaluation Committee approvals were obtained prior to dissemination of the survey. An 80% response rate was required including opt out responses such as 1) I do not take care of patients; 2) I do not take care of patients with this condition and 3) other reason. Responders were tracked (with actual responses being anonymous) and non-responders were sent regular reminder e-mails until 80% response rate was achieved. Respondents were asked to consider patients with SJIA only, as other categories of JIA were excluded. The survey had three broad sections: a) demographic information (years of practice post-fellowship and number of hours/week spent in clinical work); b) physicians’ opinions on clinical inactive disease (CID) in SJIA and c) existing practices when withdrawing medications in SJIA during CID or clinical remission on medications (CRM). The American College of Rheumatology or Wallace criteria define CID as satisfying all of the following: absence of active arthritis, uveitis and fever/ rash/ serositis/ splenomegaly/ lymphadenopathy due to JIA, normal inflammatory markers, physician global assessment 0 and ≤ 15 min morning stiffness [3]. CRM is defined as presence of CID for at least six continuous months on medication [3].

Multiple-choice questions were used and branching logic was introduced for several questions to facilitate and tailor participant responses. For certain fields free text was allowed, for example if participants did not agree with Wallace definitions for CID in SJIA. When determining what factors play a role in the decision to withdraw/ taper therapy, participants were given a list of factors (generated at 2016 SJIA workgroup meeting) and asked to rank the top five factors and quantify the importance of each (very important, moderately important, somewhat important, less important, unimportant). The section on withdrawal of medications in the survey focused on decisions around individual medication withdrawal including prednisone, methotrexate, anakinra, canakinumab, rilonacept, tocilizumab and combination therapy withdrawal. Descriptive statistics including percentages were used to analyze the data.

## Results

### Demographic results

Eighty-six of the 100 surveyed CARRA voting physician members completed the survey (86% response rate) including seven members who opted out (4 not involved in clinical care, 3 not involved SJIA patient care). This amounted to total 76 (N) responses for the survey. Respondents were not required to answer every question hence incomplete responses lead to fewer numbers (n) in each question. The majority of respondants were experienced physicians with > 10 years in practice post-fellowship 44% (*n* = 33), 28% each with 5–10 years in practice and < 5 years in practice (*n* = 21).

### Definition of inactive disease

Majority (88%, *n* = 65) agreed with current criteria for CID in SJIA. Stated reasons for dissent included adding ferritin to the definition, removing rash and uveitis, extending duration of morning stiffness, changing duration required for CID, imprecision of physician global score and lack of patient/parent-reported outcomes. Ninety-three percent (*n* = 67) agreed with current definition for CRM in SJIA. Disagreement was due to preference for 1 year of inactive disease to meet CRM. Most felt it was necessary to meet CRM (78%, *n* = 58) before tapering medications other than steroids. Those who did not agree felt the clinical significance of CRM was not clear and 6 months was too long to wait before tapering therapy.

The top five factors rated very important in decisions regarding reduction or withdrawal of disease modifying anti-rheumatic drug (DMARD) or biologic therapy for patients with SJIA that had discontinued steroids included: 1) past failure of medication taper (65.3%), 2) toxicity/side effects/tolerance of medications (64.9%), 3) time maintained in inactive disease (52%), 4) history of macrophage activation syndrome (MAS) (49.3%) and 5) number of previous flares (45.2%) (Table 1). An overwhelming 64% (*n* = 47) of respondents never (*n* = 14) or seldom (*n* = 33) used imaging to determine whether to reduce/ stop methotrexate or biologic therapy. Of those who used imaging in their decision-making tools (used seldom = 33, used sometimes *n* = 21 and used often *n* = 5), most used magnetic resonance imaging (83%, *n* = 49), ultrasound (59%, *n* = 35) and radiographic studies (22%, *n* = 13) (respondents were allowed to select more than one option for this question). Seventy-one percent (*n* = 52) did not use specific patient or parent reported outcomes in their decision making for taper/ withdrawal of methotrexate or biologic therapy. Of the 29% of physicians who used patient/ parent reported outcomes, majority used the parent/patient global assessment of disease activity (*n* = 20, 95%). Others used pain score (*n* = 11, 52%), functional scores such as the Child Health Assessment Questionnaire (CHAQ) (n = 13, 62%) and Pediatric Rheumatology Quality of Life Scale (PRQL) (*n* = 1, 5%).

**Table 1:** Factors rated important in physician decisions regarding withdrawal of medications in inactive disease for systemic juvenile idiopathic arthritis (top 5 listed very important are bolded)

Factor (*N* = 73)	Very Important n (%)	Moderately Important n (%)	Somewhat Important n (%)	Less Important n (%)	Un- important n (%)
Patient/family preference	15 (21)	29 (40)	22 (30)	7 (9)	0
^a^Toxicity/side effects/tolerance of medications	48 (65)	21 (28)	5 (7)	0	0
Poor adherence to medications	14 (19)	34 (47)	19 (26)	6 (8)	0
Younger age at diagnosis	3 (4)	8 (11)	23 (32)	31 (42)	8 (11)
^**b**^Duration of disease	8 (12)	35 (49)	13 (18)	13 (18)	2 (3)
Time maintained in inactive disease	38 (52)	29 (40)	5 (7)	1 (1)	0
^**c**^Amount of time to achieve inactive disease	24 (32)	36 (49)	12 (16)	2 (3)	0
^**c**^Total number of DMARDs/ biologics used since diagnosis	19 (26)	29 (38)	13 (18)	11 (15)	2 (3)
Presence of JIA associated damage (joint or growth)	15 (21)	40 (56)	12 (16)	4 (6)	2 (3)
History of MAS	36 (50)	27 (37)	8 (11)	1 (1)	1 (1)
^**d**^History of previous cardiac or pulmonary involvement	29 (41)	24 (33)	15 (21)	3 (4)	1 (1)
History of previous ICU admission	20 (27)	34 (47)	14 (19)	3 (4)	2 (3)
Number of previous flares	33 (45)	25 (34)	13 (18)	2 (3)	0
^d^Past failure of medication taper	47 (65)	20 (28)	5 (7)	0	0
Anticipated social or environmental changes	1 (1)	30 (42)	27 (37)	14 (19)	1 (1)

Abbreviations: *N* = Total number of responses for the specific factor

^a^ *N* = 74, ^b^ *N* = 71, ^c^ *N* = 74 ^d^ *N* = 72

Other factors that physicians listed included: making plans for parenthood or discovery of pregnancy with intention to maintain pregnancy, new diagnoses, monitoring ESR/CRP/ferritin, financial considerations including coverage and amount of out-of-pocket payments, access to care, and season

Use of CARRA CTP: The majority (76%, *n* = 55) follow the SJIA CARRA consensus treatment plans (CTPs) [4]. Six percent (n = 4) follow the CARRA SJIA CTPs all the time, while 71% (*n* = 51) follow them majority of the time, 18% (n = 13) follow them < 50% of time and 6% (n = 4) never follow them.

Sixty-one percent (*n* = 45) do not use glucocorticoid SJIA CTP and amongst those (38%, *n* = 28) that use this CTP, 65% (*n* = 48) follow the taper plan outlined in this publication [4]. Factors ranked important regarding decisions on tapering prednisone included: severity of disease (89%), side effects to prednisone (85%), prior success with tapering prednisone (47%) and patient preference (38%).

Nineteen percent (*n* = 14) did not use the methotrexate SJIA CTP plan.

With respect to anti-IL1 therapy usage, 5% never used anakinra, 15% never used canakinumab and 69% never used rilonacept for the treatment of SJIA. In contrast, only 3% never used tocilizumab for the treatment of SJIA.

**Fig. 1:**
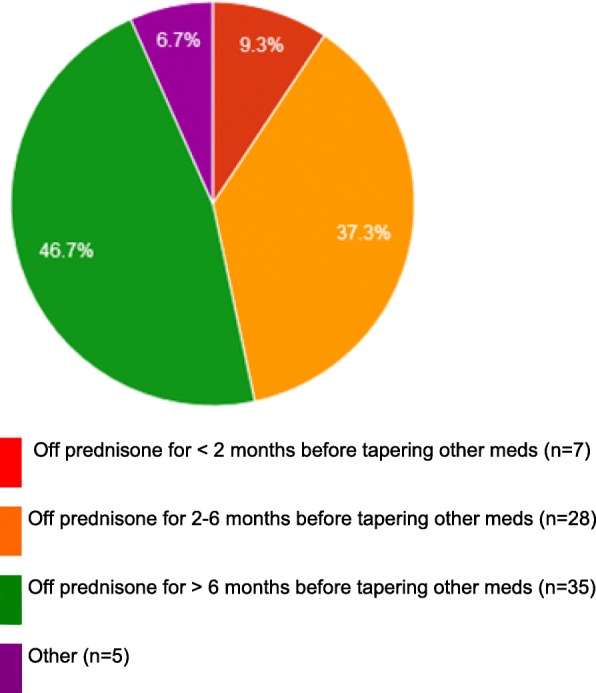
Duration in months to tapering other medication(s) after patient has successfully discontinued glucocorticoids

Taper of specific medications: All members (100%, *n* = 74) weaned steroids first in SJIA patients on combination therapy, and none started a taper of remaining medications immediately after discontinuing prednisone. Figure 1 depicts the duration to tapering other medications after the patient has successfully discontinued glucocorticoids.

Use and tapering of methotrexate and biologic medications in SJIA patients in CID or CRM varied widely across respondents (Table 2). For canakinumab and tocilizumab, 82 and 94% respectively favor increasing the interval between injections or infusions rather that reducing the dose of each injection or infusion.

**Table 2:** Methotrexate and biologic use and tapering in SJIA: physician practices, CARRA workgroup survey

Medication	Never use this CTP % (n)	Stop immediately % (n)	Taper over weeks to < 2 months % (n)	Taper over 2–6 months % (n)	Taper over > 6 months % (n)
Methotrexate	19 (14)	7 (5)	4 (3)	35 (26)	35 (26)
Anakinra	5.3 (4)	9.3 (7)	25.3 (19)	38.7 (29)	21.3 (16)
Canakinumab	14.7 (11)	12 (9)	4 (3)	36 (27)	33.3 (25)
Rilonacept	69.3 (52)	2.7 (2)	2.7 (2)	16 (12)	(9.3 (7)
Tocilizumab	2.7 (2)	4 (3)	6.7 (5)	46.7 (35)	40 (30)

Abbreviation: *SJIA* Systemic juvenile idiopathic arthritis, *CARRA* Childhood Arthritis and Rheumatology Research Alliance, *CTP* Consensus treatment plan [4]

In patients on combination therapy with methotrexate and biologics, 59% tapered methotrexate first, 9% tapered or withdrew the biologic agent first, while most others (32%) considered patient/family preference, adverse effects, lifestyle issues, adherence and response to medication amongst other factors to guide their choice.

Most physicians follow their SJIA patients closely after withdrawal of medications such as every month (43%), and every 2–3 months (54%).

## Discussion

While the introduction of cytokine-directed therapy targeting IL-1 and IL-6 has revolutionized the treatment of SJIA, little is known regarding the approach to tapering and stopping such therapy. This CARRA survey of pediatric rheumatology providers offers the first systematic description of clinical practice patterns for medication withdrawal. A first key finding of this survey is the demonstrated agreement and consensus amongst providers in using the existing Wallace criteria for determining inactive disease and remission states in children with SJIA [3]. With the exception of steroids and non-steroidal anti-inflammatory medication withdrawal, most providers wait for clinical remission on medications to start tapering or withdrawing other medications. Since publication of the CARRA SJIA CTP plans [4] an overwhelming majority of providers have adopted these in their current practices and not surprisingly, 71% did not use the glucocorticoid CTP likely reflecting the wide acceptance of biologic use in early presentations of SJIA [5, 6]. The hesitancy to use glucocorticoid CTP plan and 100% consensus on tapering glucocorticoids first when used likely also reflect an attempt by treating providers to limit use of steroids with their expected side effect profiles [7]. This is exemplified by the fact that 85% of respondents listed side effects from prednisone as an important factor in decision-making regarding tapering of prednisone. The lack of use of patient reported outcomes or imaging in 71 and 65% of physicians respectively might reflect the lack of current SJIA specific patient reported outcomes and the lack of readily available imaging, additional cost implications or variable expertise for use of musculoskeletal ultrasound across centers. Despite the lack of use of specific patient reported outcomes most physicians take into account patient/ family preferences via a process of shared decision making as implied by the fact that 61% of respondents ranked patient or family preference in withdrawal of medications as a very or moderately important factor in their decision making process.

The second key finding of this study is the considerable variability amongst pediatric rheumatology providers in decisions on which medication to taper next when patients are on combination therapy, how long to wait before tapering of other medications after discontinuing prednisone, and over what duration to taper medications. This likely is due to the paucity of current evidence or biomarkers to guide physicians on best strategies for withdrawal of medications. A randomized, prospective trial of 364 children with JIA treated with methotrexate (10% with SJIA) examined whether longer time in CRM prevented flares after withdrawal of medication [8]. Here, continuing therapy for 12 months versus 6 months after achieving inactive disease did not significantly improve the overall flare rate. A more recent retrospective study of 1514 patients with JIA (only 4% with SJIA) has challenged this, finding flare was indeed a function of time in CRM, and that patients with inactive disease > 12 months had significantly lower flare rates [9]. There is similarly substantial interest in biomarkers to stratify patients for risk of flare upon medication withdrawal. The above study by Foell et al. did find that lower serum S100A8/A9 (calprotectin; MRP8/14) levels were associated with lower risk of flare over the subsequent 3 months [8]. Further work specifically in SJIA patients found that S100A8/A9 levels correlated with disease activity, and that higher levels during CRM predicted disease flares [10].

SJIA itself is a heterogeneous disease with certain subsets of patients following a monophasic course and others following a more persistent or relapsing course. Clinical features at presentation or during disease course are unable to predict which path an individual patient will follow; hence physicians are left to their own judgements when it comes to withdrawing medications often needing to balance the side effects of continued exposure to medications with the risk of disease flare on withdrawal of these drugs. Some physicians may taper medications based on the half life of drugs used. Survey respondents noted that in patients on canakinumab, rilonacept and tocilizumab, most would prefer increasing the time between injections or infusions rather than reducing the dose likely reflecting the preference in patients for fewer injections/ infusions. Results from the long-term extension phase III trials of canakinumab reported that 25% (*n* = 44) were able to reduce the dose of canakinumab to 2 mg/kg and 59% (*n* = 26) of these were able to maintain this reduced doing schedule for 25 months. Eighteen patients eventually flared with the tapered schedule and required either increase in dose back to 4 mg/kg of canakinumab or additional medications [11]. More recent data from the same group examined the efficacy and safety of two different canakinumab tapering regimens (3-step dose taper of 2 mg/kg/q 4 weeks then 1 mg/kg/q 4 weeks then stop versus interval increase in dose 4 mg/kg q 8 weeks then 4 mg/kg/q 12 weeks then stop). Patients were eligible for taper if they were off methotrexate and corticosteroids in inactive disease for 24 months. While several patients were able to taper to an extent on both regimens (71% able to reduce dose to 2 mg/kg q 4 weeks, 84% at 4 mg/kg q 8 weeks) only 33% (*n* = 25/75)were able to discontinue canakinumab and maintain without flare for 24 weeks [12]. Data from the TENDER clinical trial (*N* = 112) studied tocilizumab taper in SJIA after 2 years of treatment and CID for 3 months by spacing the infusions from every 2 weeks to 3 weeks then 4 weeks then stopping infusions (35%, *n* = 39 were eligible for taper). At the time of last data review (May 2014), 7 patients were able to discontinue tocilizumab while 2 patients had to revert back to regular q 2 week doing schedule [13].

Simonini et al. studied remission rates after withdrawal of biologic agents in 135 JIA children (8% of who had SJIA) and found that likelihood of staying in remission was higher for SJIA patients [14]. Similarly, in the ReACCH- Out Canadian JIA cohort (*N* = 1104) about 47% of SJIA patients (*n* = 76) achieved remission in 5 years [6]. Shoop-Worrall et al. also noted in their systematic review that rates of CID or remission in SJIA varied widely ranging from 0 to 100% thus reflecting the lack of current knowledge in what drives and/ or sustains remission or CID in SJIA [15].

Strengths of the study include a high response rate of 86%, with a majority of participants being seasoned pediatric rheumatology practitioners (72% being > 5 years into practice of pediatric rheumatology). This suggests good representation across a large sample of the CARRA network. Limitations of the study include that results were self-reported thus introducing recall bias. Many questions in the survey were closed ended and had distinct options to choose from thus lowering the validity rate of the survey. We are limited by the heterogeneity of surveyed pediatric rheumatologists including the fact that 28% of respondents had < 5 years of pediatric rheumatology experience and differences between access to medication across different centers, especially with regard to biological drugs.

## Conclusion

Most CARRA members are using existing consensus treatment plans for SJIA and agree with validated definitions of CID and CRM. There was also agreement to taper steroids first, but there was considerable variability for all other medications.

## Additional file

Additional file 1: CARRA SJIA Inactive Disease and Withdrawal of Medications Survey. (DOCX 379 kb)

## Data Availability

All are included in the tables and manuscript.

## Abbreviations

CARRA: Childhood Arthritis and Rheumatology Research Alliance
CID: Clinical inactive disease
CRM: Clinical remission on medications
CTP: Consensus treatment plan
IRB: Institutional review board
SJIA: Systemic Juvenile Idiopathic Arthritis
