# Supplementary material for: Physician practices for withdrawal of medications in inactive systemic juvenile arthritis, Childhood Arthritis and Rheumatology Research Alliance (CARRA) survey
Source: Pediatr Rheumatol Online J. 2019 Jul 22;17:48. doi: 10.1186/s12969-019-0342-5 (PMC6647107; doi:10.1186/s12969-019-0342-5)
Supplement: Supplementary file 1 — CARRA SJIA Inactive Disease and Withdrawal of Medications Survey. (DOCX 379 kb) [file 12969_2019_342_MOESM1_ESM.docx]

| **CARRA SJIA Inactive Disease and Withdrawal of Medications Survey**   | |
| --- | --- |
| \| Thank you for taking the time to do this survey. If you would like to opt out of this survey you may do so now by choosing one of these responses: \| [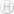](javascript:;) [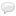](javascript:;) \| \| --- \| --- \| |   I do not take care of patients  I do not take care of patients with this condition  [reset](javascript:;) |
| \| Please specify other. \| [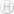](javascript:;) [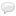](javascript:;) \| \| --- \| --- \| |   [Expand](javascript:;) |
| **Please review the attached information sheet prior to beginning the survey.**  Attachment:   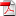[INFORMATION SHEET 2-14-2017.pdf](https://redcap.iths.org/redcap_v8.11.9/DataEntry/file_download.php?pid=7261&page=carra_sjia_inactive_disease_and_withdrawal_of_medi&type=attachment&doc_id_hash=95d905c27b59c5075a4d0b489af34adf7fcb4d17&instance=1&id=253319)  (0.26 MB) | |
| **Purpose: The purpose of this survey is to determine a modification of the definition for inactive disease (if any is needed) for Systemic Juvenile Idiopathic Arthritis (SJIA) to be used for a future consensus treatment plan (CTP) for withdrawal of medication. Additionally, we will determine existing practices amongst pediatric rheumatologists regarding withdrawing medications in SJIA during inactive disease (ID) or clinical remission on medications (CRM).  Section 1: Inactive Disease  Wallace criteria:  Clinical Inactive Disease (CID): *No joints with active arthritis *No fever, rash, serositis, splenomegaly, or generalized lymphadenopathy attributable to JIA *No active uveitis (Standardization of Uveitis Nomenclature {SUN} criteria) *Best possible physician's global assessment of disease activity score (for example, 0 on a 0-10 Visual Analog Scale) *Normal ESR and/or CRP; if elevated, not attributable to JIA *Duration of morning stiffness â‰¤15 minutes  Clinical Remission on Medication (CRM): The criteria for inactive disease must be met for a minimum of 6 continuous months while the patient is on medication.** | |
| \| Do you agree with the definition of Clinical Inactive Disease in SJIA as stated above? \| [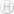](javascript:;) [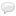](javascript:;) \| \| --- \| --- \| |   Yes  No  [reset](javascript:;) |
| \| If no, why not? \| [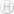](javascript:;) [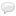](javascript:;) \| \| --- \| --- \| |   [Expand](javascript:;) |
| \| Do you agree with the definition of Clinical Remission on Medications (CRM) in SJIA as stated above? \| [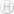](javascript:;) [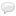](javascript:;) \| \| --- \| --- \| |   Yes  No  [reset](javascript:;) |
| \| If no, why not? \| [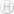](javascript:;) [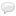](javascript:;) \| \| --- \| --- \| |   [Expand](javascript:;) |
| \| Do you think it's necessary to meet CRM criteria before considering a taper of medications (other than steroids)? \| [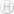](javascript:;) [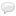](javascript:;) \| \| --- \| --- \| |   Yes  No  [reset](javascript:;) |
| \| If no, why not? \| [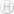](javascript:;) [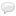](javascript:;) \| \| --- \| --- \| |   [Expand](javascript:;) |
| **4. In general, how important are the following factors in deciding whether to reduce/stop DMARD/biologic therapy for patients with systemic JIA (off steroids):** | |
| \|  \| **Very important** \| **Moderately important** \| **Somewhat important** \| **Less important** \| **Unimportant** \| \| --- \| --- \| --- \| --- \| --- \| --- \| | |
| ****   \| \| Patient/family preference \| [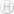](javascript:;) [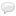](javascript:;) \| \| --- \| --- \| \|  \|  \|  \|  \|  \| \| --- \| --- \| --- \| --- \| --- \| --- \| --- \| --- \|   [reset](javascript:;) | |
| ****   \| \| Toxicity/side effects/tolerance of medications \| [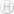](javascript:;) [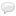](javascript:;) \| \| --- \| --- \| \|  \|  \|  \|  \|  \| \| --- \| --- \| --- \| --- \| --- \| --- \| --- \| --- \|   [reset](javascript:;) | |
| ****   \| \| Poor adherence to medications \| [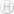](javascript:;) [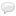](javascript:;) \| \| --- \| --- \| \|  \|  \|  \|  \|  \| \| --- \| --- \| --- \| --- \| --- \| --- \| --- \| --- \|   [reset](javascript:;) | |
| ****   \| \| Younger age at diagnosis \| [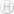](javascript:;) [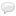](javascript:;) \| \| --- \| --- \| \|  \|  \|  \|  \|  \| \| --- \| --- \| --- \| --- \| --- \| --- \| --- \| --- \|   [reset](javascript:;) | |
| ****   \| \| Duration of disease \| [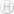](javascript:;) [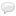](javascript:;) \| \| --- \| --- \| \|  \|  \|  \|  \|  \| \| --- \| --- \| --- \| --- \| --- \| --- \| --- \| --- \|   [reset](javascript:;) | |
| ****   \| \| Time maintained in inactive disease \| [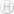](javascript:;) [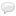](javascript:;) \| \| --- \| --- \| \|  \|  \|  \|  \|  \| \| --- \| --- \| --- \| --- \| --- \| --- \| --- \| --- \|   [reset](javascript:;) | |
| ****   \| \| Amount of time to achieve inactive disease \| [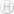](javascript:;) [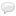](javascript:;) \| \| --- \| --- \| \|  \|  \|  \|  \|  \| \| --- \| --- \| --- \| --- \| --- \| --- \| --- \| --- \|   [reset](javascript:;) | |
| ****   \| \| Total number of DMARDs/biologics used since diagnosis \| [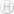](javascript:;) [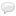](javascript:;) \| \| --- \| --- \| \|  \|  \|  \|  \|  \| \| --- \| --- \| --- \| --- \| --- \| --- \| --- \| --- \|   [reset](javascript:;) | |
| ****   \| \| Presence of JIA associated damage (joint or growth) \| [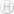](javascript:;) [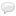](javascript:;) \| \| --- \| --- \| \|  \|  \|  \|  \|  \| \| --- \| --- \| --- \| --- \| --- \| --- \| --- \| --- \|   [reset](javascript:;) | |
| ****   \| \| History of MAS \| [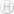](javascript:;) [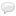](javascript:;) \| \| --- \| --- \| \|  \|  \|  \|  \|  \| \| --- \| --- \| --- \| --- \| --- \| --- \| --- \| --- \|   [reset](javascript:;) | |
| ****   \| \| History of previous cardiac or pulmonary involvement \| [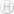](javascript:;) [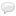](javascript:;) \| \| --- \| --- \| \|  \|  \|  \|  \|  \| \| --- \| --- \| --- \| --- \| --- \| --- \| --- \| --- \|   [reset](javascript:;) | |
| ****   \| \| History of previous ICU admission \| [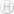](javascript:;) [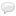](javascript:;) \| \| --- \| --- \| \|  \|  \|  \|  \|  \| \| --- \| --- \| --- \| --- \| --- \| --- \| --- \| --- \|   [reset](javascript:;) | |
| ****   \| \| Number of previous flares \| [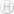](javascript:;) [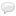](javascript:;) \| \| --- \| --- \| \|  \|  \|  \|  \|  \| \| --- \| --- \| --- \| --- \| --- \| --- \| --- \| --- \|   [reset](javascript:;) | |
| ****   \| \| Past failure of medication taper \| [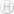](javascript:;) [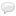](javascript:;) \| \| --- \| --- \| \|  \|  \|  \|  \|  \| \| --- \| --- \| --- \| --- \| --- \| --- \| --- \| --- \|   [reset](javascript:;) | |
| \| \| Anticipated social or environmental changes \| [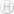](javascript:;) [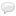](javascript:;) \| \| --- \| --- \| \|  \|  \|  \|  \|  \| \| --- \| --- \| --- \| --- \| --- \| --- \| --- \| --- \|   [reset](javascript:;) | |
| \| \| Other \| [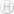](javascript:;) [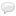](javascript:;) \| \| --- \| --- \| \|  \|  \|  \|  \|  \| \| --- \| --- \| --- \| --- \| --- \| --- \| --- \| --- \|   [reset](javascript:;) | |
| \| Briefly, what other factor is important to you in deciding whether to reduce/stop therapy? \| [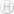](javascript:;) [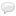](javascript:;) \| \| --- \| --- \| | [Expand](javascript:;) |
| **4b. Please rank top 5 factors from 1-5 (most important to least important) in deciding whether or not to reduce/stop DMARD/biological therapy for patients with systemic JIA.** | |
| \| (One selection allowed per column) \| **1** \| **2** \| **3** \| **4** \| **5** \| \| --- \| --- \| --- \| --- \| --- \| --- \| | |
| \| \| Patient/family preference \| [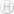](javascript:;) [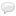](javascript:;) \| \| --- \| --- \| \|  \|  \|  \|  \|  \| \| --- \| --- \| --- \| --- \| --- \| --- \| --- \| --- \|   [reset](javascript:;) | |
| \| \| Toxicity/side effects/tolerance of medications \| [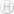](javascript:;) [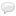](javascript:;) \| \| --- \| --- \| \|  \|  \|  \|  \|  \| \| --- \| --- \| --- \| --- \| --- \| --- \| --- \| --- \|   [reset](javascript:;) | |
| \| \| Poor adherence to medications \| [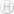](javascript:;) [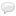](javascript:;) \| \| --- \| --- \| \|  \|  \|  \|  \|  \| \| --- \| --- \| --- \| --- \| --- \| --- \| --- \| --- \|   [reset](javascript:;) | |
| \| \| Younger age at diagnosis \| [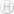](javascript:;) [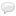](javascript:;) \| \| --- \| --- \| \|  \|  \|  \|  \|  \| \| --- \| --- \| --- \| --- \| --- \| --- \| --- \| --- \|   [reset](javascript:;) | |
| \| \| Duration of disease \| [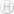](javascript:;) [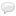](javascript:;) \| \| --- \| --- \| \|  \|  \|  \|  \|  \| \| --- \| --- \| --- \| --- \| --- \| --- \| --- \| --- \|   [reset](javascript:;) | |
| \| \| Time maintained in inactive disease \| [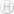](javascript:;) [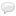](javascript:;) \| \| --- \| --- \| \|  \|  \|  \|  \|  \| \| --- \| --- \| --- \| --- \| --- \| --- \| --- \| --- \|   [reset](javascript:;) | |
| \| \| Amount of time to achieve inactive disease \| [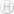](javascript:;) [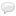](javascript:;) \| \| --- \| --- \| \|  \|  \|  \|  \|  \| \| --- \| --- \| --- \| --- \| --- \| --- \| --- \| --- \|   [reset](javascript:;) | |
| \| \| Total number of DMARDs/biologics used since diagnosis \| [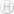](javascript:;) [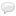](javascript:;) \| \| --- \| --- \| \|  \|  \|  \|  \|  \| \| --- \| --- \| --- \| --- \| --- \| --- \| --- \| --- \|   [reset](javascript:;) | |
| \| \| Presence of JIA associated damage (joint or growth) \| [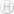](javascript:;) [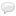](javascript:;) \| \| --- \| --- \| \|  \|  \|  \|  \|  \| \| --- \| --- \| --- \| --- \| --- \| --- \| --- \| --- \|   [reset](javascript:;) | |
| \| \| History of MAS \| [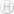](javascript:;) [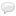](javascript:;) \| \| --- \| --- \| \|  \|  \|  \|  \|  \| \| --- \| --- \| --- \| --- \| --- \| --- \| --- \| --- \|   [reset](javascript:;) | |
| \| \| History of previous cardiac or pulmonary involvement \| [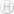](javascript:;) [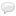](javascript:;) \| \| --- \| --- \| \|  \|  \|  \|  \|  \| \| --- \| --- \| --- \| --- \| --- \| --- \| --- \| --- \|   [reset](javascript:;) | |
| \| \| History of previous ICU admission \| [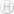](javascript:;) [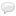](javascript:;) \| \| --- \| --- \| \|  \|  \|  \|  \|  \| \| --- \| --- \| --- \| --- \| --- \| --- \| --- \| --- \|   [reset](javascript:;) | |
| \| \| Number of flares when disease was inactive \| [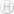](javascript:;) [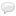](javascript:;) \| \| --- \| --- \| \|  \|  \|  \|  \|  \| \| --- \| --- \| --- \| --- \| --- \| --- \| --- \| --- \|   [reset](javascript:;) | |
| \| \| Past failure of medication taper \| [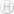](javascript:;) [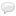](javascript:;) \| \| --- \| --- \| \|  \|  \|  \|  \|  \| \| --- \| --- \| --- \| --- \| --- \| --- \| --- \| --- \|   [reset](javascript:;) | |
| \| \| Anticipated social or environmental changes \| [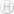](javascript:;) [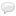](javascript:;) \| \| --- \| --- \| \|  \|  \|  \|  \|  \| \| --- \| --- \| --- \| --- \| --- \| --- \| --- \| --- \|   [reset](javascript:;) | |
| \| \| Other (as specified in question 4) \| [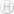](javascript:;) [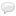](javascript:;) \| \| --- \| --- \| \|  \|  \|  \|  \|  \| \| --- \| --- \| --- \| --- \| --- \| --- \| --- \| --- \|   [reset](javascript:;) | |
| \| Do you use imaging to determine whether to reduce/stop methotrexate/biologic therapy for patients with systemic JIA? \| [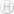](javascript:;) [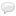](javascript:;) \| \| --- \| --- \| | Never  Seldom  Sometimes  Often  Always  [reset](javascript:;) |
| \| Which imaging modalities do you use to guide your decision? (You may select more than one option.) \| [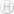](javascript:;) [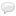](javascript:;) \| \| --- \| --- \| | MRI  Ultrasound  Radiograph |
| \| Beyond discussing with the patient/family, do you use specific patient/parent-reported outcomes to decide whether to reduce/stop methotrexate/biologic therapy? \| [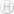](javascript:;) [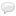](javascript:;) \| \| --- \| --- \| | Yes  No  [reset](javascript:;) |
| \| If yes for #6, which patient/parent-reported outcome(s)? (You may select more than one option.) \| [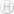](javascript:;) [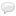](javascript:;) \| \| --- \| --- \| | Parent/patient assessment of disease activity (global score)  Pain score  Functional score (e.g. Childhood Health Assessment Questionnaire {CHAQ})  Quality of life score (e.g. Pediatric Rheumatology Quality of Life Scale {PRQL})  Other |
| \| What other patient/parent-reported outcome? \| [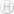](javascript:;) [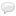](javascript:;) \| \| --- \| --- \| | [Expand](javascript:;) |
| **Section 2: Withdrawal of Medications  Please respond to the following case scenarios/questions pertaining to withdrawal of medications in SJIA** | |
| \| Please choose a case definition of SJIA for the withdrawal (CTP). (Refer to DeWitt et al 2012 Table 1) \| [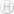](javascript:;) [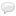](javascript:;) \| \| --- \| --- \| | ILAR definition  Operational case definition of SJIA (As defined by DeWitt et al 2012 Table 1)  SJIA as diagnosed by attending pediatric rheumatologist  Include all of the above options  [reset](javascript:;) |
| Attachment:   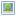[DeWitt et al 2012 Table 1.JPG](https://redcap.iths.org/redcap_v8.11.9/DataEntry/file_download.php?pid=7261&page=carra_sjia_inactive_disease_and_withdrawal_of_medi&type=attachment&doc_id_hash=93b12867863a8e8bcd5a0cabdece885f1a77a97a&instance=1&id=253320)  (0.1 MB) | |
| \| In general do you follow the current consensus treatment plans (CTPs) developed for treatment of SJIA? (use articles below) \| [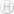](javascript:;) [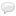](javascript:;) \| \| --- \| --- \| | Yes, always  Yes, majority of the time (>50%)  Yes, minority of the time (< 50%)  Never  [reset](javascript:;) |
| Attachment:   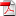[carra_frost_pc_12sep2016.pdf](https://redcap.iths.org/redcap_v8.11.9/DataEntry/file_download.php?pid=7261&page=carra_sjia_inactive_disease_and_withdrawal_of_medi&type=attachment&doc_id_hash=23419ef7177f4fe0f3d1a1ff1baacc3a2891c849&instance=1&id=253321)  (0.08 MB) | |
| \| Please specify why not. \| [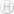](javascript:;) \| \| --- \| --- \| | [Expand](javascript:;) |
| \| Assuming you have selected the glucocorticoid only arm for your SJIA patient and they meet the current working definition of ID/CRM are you agreeable to following the taper plan outlines in the CTP for JIA? (see DeWitt et al.2012 SJIA CTP steroid taper below) \|  \| \| --- \| --- \| | Yes  No  I never use the glucocorticoid only CTP  [reset](javascript:;) |
| Attachment:   [DeWitt et al 2012.SJIA CTP steroid taper.pdf](https://redcap.iths.org/redcap_v8.11.9/DataEntry/file_download.php?pid=7261&page=carra_sjia_inactive_disease_and_withdrawal_of_medi&type=attachment&doc_id_hash=e00c69668cb50850e991ab778c6a3d71de618688&instance=1&id=253322)  (0.17 MB) | |
| \| Specify why not and your usual approach. \|  \| \| --- \| --- \| | [Expand](javascript:;) |
| \| You have a patient on combination therapy with prednisone and another agent (e.g. methotrexate, IL - 1 inhibitor, tocilizumab) who is now doing well and you are considering reducing therapy. Do you usually taper prednisone first? \|  \| \| --- \| --- \| | Yes  No  [reset](javascript:;) |
| \| Please specify why. \|  \| \| --- \| --- \| | [Expand](javascript:;) |
| \| Do you begin tapering prednisone based on recommended guidelines (per glucocorticoid only arm) once in ID/CRM? \|  \| \| --- \| --- \| | Yes  No  [reset](javascript:;) |
| \| Please specify why not and what your usual approach is to taper prednisone. \|  \| \| --- \| --- \| | [Expand](javascript:;) |
| \| What factors influence your decision when considering how fast you taper prednisone? (You may select more than one option.) \|  \| \| --- \| --- \| | Patient preference  Side effects to prednisone  Prior success with tapering of prednisone in other patients  Severity of disease  Other |
| \| Please specify: \|  \| \| --- \| --- \| | [Expand](javascript:;) |
| \| Your SJIA patient on combination therapy has successfully tapered and discontinued prednisone. How long do you usually wait before tapering other medication(s)? (e.g. methotrexate, IL - 1 inhibitor, tocilizumab) \|  \| \| --- \| --- \| | Start taper immediately after stopping prednisone  Wait off prednisone for < 2 months before tapering other medication  Wait off prednisone for 2-6 months before tapering other medication  Wait off prednisone for > 6 months before tapering other medication  Other  [reset](javascript:;) |
| \| Please specify other. \|  \| \| --- \| --- \| | [Expand](javascript:;) |
| \| You have a SJIA patient on only methotrexate who is in ID/CRM and off prednisone. How quickly would you taper methotrexate? \|  \| \| --- \| --- \| | Stop immediately  Taper over weeks to < 2 months  Taper over 2-6 months  Taper over >6 months  I never use the methotrexate only CTP  [reset](javascript:;) |
| \| You have a SJIA patient on only anakinra who is in ID/CRM and off prednisone. How quickly would you taper anakinra (Half life: 4-6 hours)? \|  \| \| --- \| --- \| | Stop immediately  Taper over weeks to < 2 months  Taper over 2-6 months  Taper over >6 months  I never use the anakinra only CTP  [reset](javascript:;) |
| \| You have a SJIA patient on only canakinumab who is in ID/CRM and off prednisone. How quickly would you taper canakinumab (Half life: â‰¥4 years: 23-26 days)? \|  \| \| --- \| --- \| | Stop immediately  Taper over weeks to < 2 months  Taper over 2-6 months  Taper over >6 months  I never use the canakinumab only CTP  [reset](javascript:;) |
| \| You have a SJIA patient on only canakinumab who is in ID/CRM and off prednisone. How would you taper canakinumab (Half life: â‰¥4 years: 23-26 days)? \|  \| \| --- \| --- \| | Decrease the dose keeping the injections at every 4 weeks  Increase the interval between injections, keeping the dose the same (e.g. q6 then q8 weeks etc.)  Other  [reset](javascript:;) |
| \| Please describe how you would taper canakinumab. \|  \| \| --- \| --- \| | [Expand](javascript:;) |
| \| You have a SJIA patient on only rilonacept who is in ID/CRM and off prednisone. How quickly would you taper rilonacept (Half life: one week)? \|  \| \| --- \| --- \| | Stop immediately  Taper over weeks to < 2 months  Taper over 2-6 months  Taper over >6 months  I never use the rilonacept only CTP  [reset](javascript:;) |
| \| You have a SJIA patient on only rilonacept who is in ID/CRM and off prednisone. How would you taper rilonacept (Half life: one week)? \|  \| \| --- \| --- \| | Decrease the dose, keeping the injections at every 4 weeks  Increase the interval between injections, keeping the dose the same (e.g. q6 then q8 weeks etc.)  Other  [reset](javascript:;) |
| \| Please describe how you would taper rilonacept. \|  \| \| --- \| --- \| | [Expand](javascript:;) |
| \| You have a SJIA patient on only tocilizumab who is in ID/CRM and off prednisone. How quickly would you taper tocilizumab (Half life: 1 week)? \|  \| \| --- \| --- \| | Stop immediately  Taper over weeks to < 2 months  Taper over 2-6 months  Taper over >6 months  I never use the tocilizumab only CTP  [reset](javascript:;) |
| \| You have a SJIA patient on only tocilizumab who is in ID/CRM and off prednisone. How would you taper tocilizumab (Half life: 1 week)? \|  \| \| --- \| --- \| | Decrease the dose, keeping the infusions at every 2 weeks  Increase the interval between infusions, keeping the dose the same (e.g. q3 then q4 weeks etc.)  Other  [reset](javascript:;) |
| \| Please describe how you would taper tocilizumab. \|  \| \| --- \| --- \| | [Expand](javascript:;) |
| \| If your patient is on combination therapy with methotrexate and a biologic (anti-IL 1 or tocilizumab) which do you stop first? \|  \| \| --- \| --- \| | Methotrexate  Biologic agent  Both at the same time  Choice depends on other factors  [reset](javascript:;) |
| \| Please list other factors. \|  \| \| --- \| --- \| | [Expand](javascript:;) |
| \| From a research perspective in an ideal situation what specimens (samples) would you like to collect after initiation of withdrawal of medication and at what frequency? (Check all that apply.) \|  \| \| --- \| --- \| | ESR  CRP  S100 proteins  Other specimens  Imaging |
| \| Specify ESR frequency in months. \|  \| \| --- \| --- \| | [Expand](javascript:;) |
| \| Specify CRP frequency in months. \|  \| \| --- \| --- \| | [Expand](javascript:;) |
| \| Specify S100 proteins frequency in months. \|  \| \| --- \| --- \| | [Expand](javascript:;) |
| \| Specify both specimens and frequency in months. \|  \| \| --- \| --- \| | [Expand](javascript:;) |
| \| Specify both type of imaging (e.g. MRI, US, X-ray) and frequency in months. \|  \| \| --- \| --- \| | [Expand](javascript:;) |
| \| In an ideal situation how frequenly would you follow up your patient after initiation of withdrawal of medication? (The SJIA CTP collected data at following time points Baseline, 2, 4, 12, 24-28 weeks with room for flexibility in the actual visit windows) \|  \| \| --- \| --- \| | Every month  Every 2-3 months  Every 3-4 months  Longer intervals  [reset](javascript:;) |
| \| Specify longer intervals. \|  \| \| --- \| --- \| | [Expand](javascript:;) |
| \| Please provide any additional comments regarding the survey. \|  \| \| --- \| --- \| | [Expand](javascript:;) |
| **Section 3: Personal Information** | |
| \| How many years have you been in practice post-fellowship or training? \|  \| \| --- \| --- \| | 0-5 years  5-10 years  >10 years  [reset](javascript:;) |
| \| How many hours do you spend per week in direct patient care? \|  \| \| --- \| --- \| |  |
